# Utilization of machine learning models and grey wolf optimization method in estimation of pharmaceutical solubility in supercritical CO_2_

**DOI:** 10.3389/fchem.2026.1876302

**Published:** 2026-08-03

**Authors:** Nidal H. Abu-Hamdeh, Abdulmalik A. Aljinaidi, Ahmed B. Khoshaim

**Affiliations:** 1 Center of Research Excellence in Renewable Energy and Power Systems/Energy Efficiency Group, Department of Mechanical Engineering, Faculty of Engineering, King Abdulaziz University, Jeddah, Saudi Arabia; 2 Department of Mechanical Engineering, Faculty of Engineering, King Abdulaziz University, Jeddah, Saudi Arabia

**Keywords:** Elastic net regression, Gaussian process regression, Orthogonal matching pursuit, solubility, supercritical CO_2_

## Abstract

Accurate prediction of solubility and solvent density in supercritical fluids is essential for the efficient design and optimization of pharmaceutical and chemical processes. In this study, three machine learning regression models—Elastic Net Regression (ENR), Orthogonal Matching Pursuit (OMP), and Gaussian Process Regression (GPR)—were developed to predict the solubility of Crizotinib and the density of supercritical carbon dioxide (ScCO_2_). The Grey Wolf Optimizer (GWO) algorithm was employed for hyperparameter tuning to enhance model performance and ensure robust generalization. Experimental data covering a temperature range of 308–338 K and a pressure range of 120–270 bar were used for model training and validation. Among the developed models, GPR demonstrated the highest predictive accuracy for both solvent density and solubility. For density prediction, GPR achieved an R^2^ value of 0.9979, a root mean square error (RMSE) of 4.36, and an average absolute relative deviation (AARD) of 0.35 percent during training. On the test set, the corresponding values were R^2^ of 0.9847, RMSE of 16.34, and AARD of 2.12 percent. For solubility prediction, the GPR model achieved an R^2^ of 0.9848, RMSE of 0.0028, and AARD of 4.97 percent on the training set, while test results showed R^2^ of 0.9831, RMSE of 0.0035, and AARD of 7.70 percent. The ENR and OMP models yielded slightly lower accuracy, confirming the nonlinear nature of the system and the effectiveness of the GPR model in capturing complex thermodynamic relationships. The analysis of model predictions revealed that solvent density increased nearly linearly with pressure and decreased with temperature, while solubility displayed a crossover trend with temperature, reflecting the characteristic behavior of supercritical fluids. Overall, the proposed GWO–GPR hybrid framework provided excellent accuracy, stability, and interpretability, demonstrating its strong potential for modeling complex thermophysical properties and supporting the design of supercritical CO_2_-based pharmaceutical processes.

## 1 Introduction

Solubility of pharmaceuticals in supercritical solvents is critical for utilization of this method in processing of powder-based pharmaceuticals. The size of drug particles can be controlled to achieve high-quality drugs with enhanced amount of solubility (Abourehab et al., 2022; Bazaei et al., 2024; Li et al., 2023). Thus, the method of supercritical-fluid processing is recognized as a useful technique to address one of the problems in pharmaceutical area, i.e., poor water solubility of medicines. On the other hand, computational modeling approach can be used to correlate the size and solubility of drug to the processing parameters such as pressure. Some thermodynamic and machine learning models have been applied so far for evaluation of solubility in supercritical CO_2_. The models investigate a solid-liquid equilibrium and estimate the solubility in the supercritical phase (Abadian et al., 2023; Obaidullah and Almehizia, 2023; Sheikhi et al., 2025; Sodeifian et al., 2024; Wang and Su, 2022).

Machine learning (ML) techniques have emerged as powerful tools in modern chemical and process engineering, offering data-driven solutions for systems characterized by nonlinear and multivariate relationships (Almehizia et al., 2023; Begum, 2023; Liu et al., 2024). In particular, the modeling of thermophysical properties such as solubility and solvent density often involves complex dependencies on temperature, pressure, and molecular interactions that are difficult to capture using conventional empirical or semi-empirical correlations. ML algorithms have demonstrated remarkable capabilities in addressing such complexities by learning from experimental data and generalizing underlying patterns without requiring explicit physical equations. Consequently, their application has expanded rapidly in fields such as phase equilibrium prediction, solvent screening, and materials design, where they enable accurate, cost-effective, and time-efficient analysis of complex systems (Abouzied et al., 2023; Atwan et al., 2025; Ghanavati et al., 2024; Zhu et al., 2021).

In the area of supercritical fluid technology, the accurate estimation of solvent density and solubility plays a critical role in the optimization of extraction, crystallization, and purification processes. Supercritical carbon dioxide (ScCO_2_), in particular, has gained wide attention due to its environmentally benign nature, moderate critical parameters, and tunable solvent power (Alsaab and Althobaiti, 2025). However, the relationship between pressure, temperature, and the resulting solvent properties in ScCO_2_ is highly nonlinear, especially near the critical point, making accurate predictive modeling a challenging task. Traditional thermodynamic equations of state often require extensive parameter fitting and still struggle to achieve satisfactory accuracy under varying operating conditions. In contrast, ML methods provide an adaptable framework capable of learning these complex dependencies directly from experimental data, improving both predictive accuracy and generalization (Alotaibi et al., 2025).

In this study, three advanced regression algorithms—Elastic Net Regression (ENR), Orthogonal Matching Pursuit (OMP), and Gaussian Process Regression (GPR) (Alsaab and Althobaiti, 2025)—were developed and optimized to predict the density of supercritical CO_2_ and the solubility of Crizotinib, a targeted anticancer drug. These models have been successfully utilized in our previous study for prediction of raloxifene solubility (Alsaab and Althobaiti, 2025). These algorithms were selected to represent diverse modeling paradigms: ENR offers a balance between L1 and L2 regularization, enhancing both feature selection and model stability; OMP provides a sparse representation by iteratively selecting the most influential features; and GPR employs a nonparametric probabilistic approach capable of modeling highly nonlinear functional relationships while quantifying prediction uncertainty. To ensure optimal performance, the hyperparameters of all models were tuned using the Grey Wolf Optimizer (GWO). This optimization strategy allows the models to achieve a balance between bias and variance, improving robustness and accuracy across both training and test datasets.

The present work contributes to the field by integrating GWO with ML regression models to enhance predictive accuracy for solvent density and solubility in supercritical CO_2_ systems. The study provides a comparative assessment of the three modeling approaches based on multiple statistical performance indicators, including the coefficient of determination (R^2^), root mean square error (RMSE), and average absolute relative deviation (AARD%). Furthermore, comprehensive analyses of the predicted trends and residuals are conducted to interpret the thermodynamic behavior of the system and identify the crossover effects that influence solubility variations with temperature and pressure. Overall, this research demonstrates the strong potential of hybrid GWO–ML frameworks, particularly GPR-based models, for accurately predicting and interpreting physicochemical properties in supercritical fluid processes, offering valuable insights for process design and optimization in pharmaceutical and chemical engineering applications.

## 2 Data analytics

The dataset utilized in this study, obtained from (Sodeifian et al., 2022), comprises recorded values of temperature, pressure, Crizotinib solubility, and the density of supercritical carbon dioxide (ScCO_2_). All these parameters were employed in developing the machine learning models through two main stages: training and validation. Temperature values were recorded at four levels (308, 318, 328, and 338 K), while pressure measurements were obtained at six discrete points (120, 150, 180, 210, 240, and 270 bar) all of which have been designed in full factorial condition. The experimental dataset was designed as a full factorial set of four temperature levels and six pressure levels, covering the investigated range of 308–338 K and 120–270 bar. The developed models therefore demonstrate robust performance and generalization within the studied temperature–pressure domain. However, extrapolation beyond this experimental range was not evaluated in the present work. For each specific combination of temperature and pressure, the corresponding solubility of Crizotinib and the density of ScCO_2_ were determined and recorded as numerical values. This structured dataset provides a comprehensive representation of the thermophysical behavior of the system across varying operational conditions, forming the basis for model development and validation.

**FIGURE 1:**
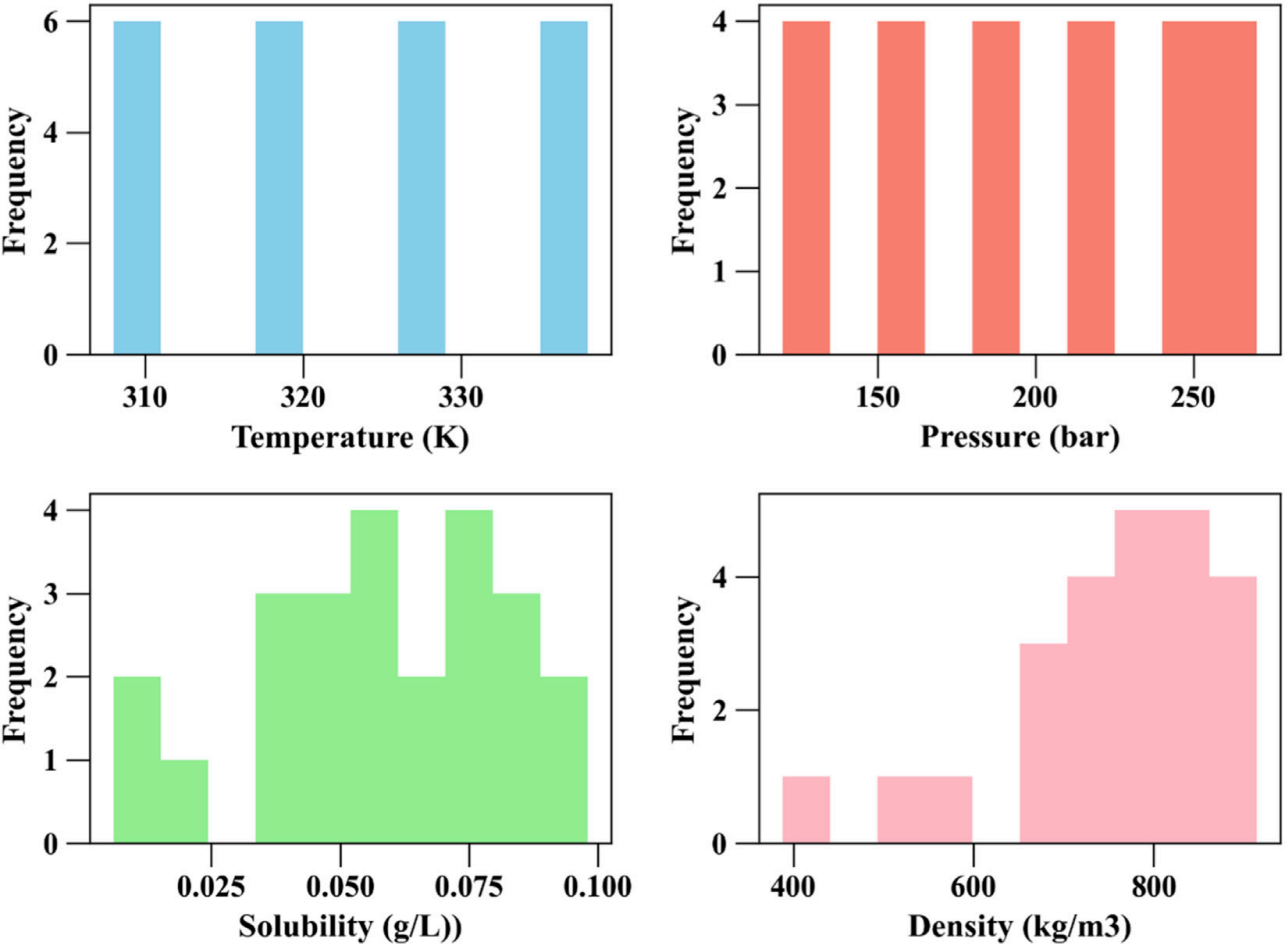
Histograms of parameter frequencies for Crizotinib solubility.

Figure 1 illustrates the frequency distributions of both input and output variables using histogram. As observed, the density variable exhibits a more pronounced skewness compared to the solubility data (Alsaab and Althobaiti, 2025). Specifically, lower solubility values occur more frequently, whereas the solvent density demonstrates a higher frequency at larger numerical values.

Prior to model training, the dataset was subjected to a preprocessing phase to enhance data quality and uniformity across all input variables. As the collected features—temperature, pressure, solubility, and CO_2_ density—exhibited different measurement scales and ranges, normalization was necessary to standardize their magnitudes. In this study, the Min–Max Normalization approach was implemented as:

$$x_{i}^{\prime }=\frac{x_{i}-x_{\min}}{x_{\max}-x_{\min}}$$

where $x_{i}$ represents an individual data point, while $x_{\min}$ and $x_{\max}$ denote the smallest and largest observed values of the respective variable. This transformation rescales all feature values within the interval [0, 1], maintaining their relative relationships while mitigating scale-induced bias. Applying this normalization ensures that each parameter contributes proportionately during model learning, thereby improving the efficiency and accuracy of the machine learning algorithms.

To ensure the integrity of the predictive results and prevent data leakage, the data normalization process was strictly partitioned. The scaling parameters (minima and maxima) were determined solely based on the training dataset. Subsequently, these fixed parameters were applied to normalize both the training and the unseen test sets. This procedure ensures that the test set remained entirely independent and that no information from the test data influenced the preprocessing or model training stages.

Following normalization, the dataset was randomly partitioned, with 80% of the data used for model training and validation, and the remaining 20% reserved for testing to evaluate predictive performance on unseen data. To ensure the robustness of the reported results and mitigate the potential influence of a single random split on model performance, this partitioning and subsequent model development, optimization, and evaluation procedure was replicated 50 times. The performance metrics presented in this study represent the average values obtained across these 50 independent runs. Importantly, the test set was maintained as completely unseen throughout the entire model optimization and training process, ensuring an unbiased evaluation of the models’ generalization capabilities.

## 3 Methodology

### 3.1 Grey wolf optimization (GWO)

The GWO is a metaheuristic technique inspired by the hierarchical structure and group hunting mechanisms observed in grey wolf populations. This approach simulates the coordinated predatory strategies of the pack, which is organized into four distinct ranks: alpha, beta, delta, and omega. In this framework, each individual wolf is represented by a set of decision variables that define its position within the multidimensional search landscape (Alsaab and Althobaiti, 2025). In GWO, the search mechanism is governed by a leadership hierarchy, wherein the positions of the alpha wolves direct the adaptive repositioning of subordinate wolves, progressively guiding the population towards optimal solutions (Alsaab and Althobaiti, 2025; Dereli, 2021; Mirjalili et al., 2014).

The position of each wolf is updated according to the following mathematical expression (Alsaab and Althobaiti, 2025; Li et al., 2019):

$$\vec{x_{i}}\left(t+1\right)=\vec{A}-\vec{C}\cdot \vec{r_{1}}\cdot \left|\vec{D}\cdot \vec{X_{i}}\left(t\right)-\vec{x_{i}}\left(t\right)\right|$$

Here, $\vec{C}$ and $\vec{A}$ stand for the coefficient vectors, $\vec{r_{1}}$ corresponds to a randomly generated vector, $\vec{D}$ represents the distance vector, and $\vec{x_{i}}\left(t\right)$ specifies the location of the *i*th wolf at the $t^{th}$ iteration (Alsaab and Althobaiti, 2025; Thajudeen et al., 2025). At every iteration, these coefficient and distance vectors are iteratively recalculated according to the wolves’ current positions, thereby enabling a dynamic balance between exploration and exploitation within the search domain (Thajudeen et al., 2025).

### 3.2 Elastic net regression (ENR)

ENR is an enhanced form of linear model that possesses the benefits of both Lasso and Ridge regularization models in data analytics applications. This approach offers a robust mechanism for simultaneous variable selection and model regularization, effectively achieving a balance between sparsity and stability in the predictive model (Alsaab and Althobaiti, 2025).

Consider a training dataset $X=\left[x_{1},x_{2},\ldots ,x_{N}\right]\in R^{N\times D}$ , where *N* stands for the number of samples and *D* represents the feature dimensionality. The objective of the ENR is to determine the optimal coefficient vector that minimizes the residual sum of squares, while concurrently encouraging sparsity and mitigating the effects of multicollinearity. The corresponding Elastic Net optimization problem can be expressed as (Alsaab and Althobaiti, 2025; Heiss et al., 2021):

$$\min_{\beta }\left(\frac{1}{2N}\sum_{i=1}^{N}\left|y_{i}-x_{i}^{T}\beta \right.{|}_{2}^{2}\right)+\lambda_{1}\left|\beta \right.{|}_{1}+\frac{\lambda_{2}}{2}\left|\beta \right.{|}_{2}^{2}$$

Here, β signifies the coefficient vector to be estimated, $y_{i}$ denotes the target value corresponding to the $i^{th}$ training sample, and $x_{i}$ refers to its associated feature vector. The notation ${\left|\cdot \right|}_{1}$ indicates the $L_{1}$ norm (Manhattan norm), while ${\left|\cdot \right|}_{2}$ signifies the $L_{2}$ norm (Euclidean norm). The regularization parameters $\lambda_{1}$ and $\lambda_{2}$ determine the strength of the $L_{1}$ and (L_2) penalties, respectively, thereby controlling the trade-off between sparsity and coefficient shrinkage in the model (Zou and Hastie, 2005). ENR uniquely integrates feature selection and regularization. The $L_{1}$ term promotes sparsity, enabling automatic variable selection and enhancing interpretability, while the $L_{2}$ term stabilizes the model by controlling coefficient magnitudes, thus mitigating multicollinearity and improving generalization (Alsaab and Althobaiti, 2025).

### 3.3 Orthogonal matching pursuit (OMP)

OMP is a sparse linear regression technique designed to approximate high-dimensional data using a limited number of predictors. The model iteratively selects features that exhibit the highest correlation with the residual of the target variable, progressively building a parsimonious representation of the response. Unlike standard regression approaches that fit all predictors simultaneously, OMP employs a greedy algorithm to add one feature at a time, ensuring orthogonality of the residuals with respect to the chosen predictors at each iteration (Alsaab and Althobaiti, 2025; Goyal and Singh, 2019).

Mathematically, OMP aims to solve the following optimization problem (Alsaab and Althobaiti, 2025; Needell and Vershynin, 2009):

$${\min_{\beta }\left|y-X\beta \right|}_{2}^{2}\text{ subject to }{\left|\beta \right|}_{0}\leq k$$

where *y* denotes the response vector, *X* indicates the matrix of input features, β stands for the coefficient vector, and *k* defines the desired sparsity level.

### 3.4 Gaussian process regression (GPR)

GPR is a nonparametric, probabilistic approach to regression that models the underlying function as a distribution over possible functions, rather than assuming a fixed functional form. This enables GPR to provide not only point predictions but also a measure of uncertainty for each prediction, making it highly valuable in scenarios requiring confidence estimation and robust modeling of complex, nonlinear relationships (Alsaab and Althobaiti, 2025; Rasmussen and Williams, 2006).

The predictive distribution for a new test input $x_{*}$ is given by a Gaussian with mean and variance computed as (Wang, 2023):

$$\mu_{*}^{\;}=k_{*}^{T}{\left(K+\sigma_{n}^{2}I\right)}^{-1}y,$$

$$\sigma_{*}^{2}=k\left(x_{*}^{\;},x_{*}^{\;}\right)-k_{*}^{T}{\left(K+\sigma_{n}^{2}I\right)}^{-1}k_{*}^{\;}$$

where K is the covariance matrix of the training data and $k_{*}$ is the covariance vector between the training and test points. These expressions provide both the expected prediction and its associated uncertainty.

The kernel function $k\left(x,x^{\prime }\right)$ plays a critical role in defining the smoothness, periodicity, and generalization ability of the model. Commonly used kernels include the Radial Basis Function (RBF), Matérn, and Rational Quadratic kernels. Hyperparameters of the kernel and noise term are typically optimized by maximizing the log marginal likelihood of the observed data.

GPR offers several advantages, such as high interpretability and the ability to capture complex, nonlinear trends with relatively few parameters. However, its computational complexity scales cubically with the number of training samples ( $O\left(N^{3}\right)$ ), which can limit its application to large datasets. Despite this, GPR remains a powerful tool for small to medium-sized regression problems where uncertainty quantification is essential (Alsaab and Althobaiti, 2025; Liu et al., 2020).

To support reproducibility, all stochastic components of the workflow (including random data splitting and model initialization/optimization) were controlled using a fixed random seed (seed = 42). Hyperparameter optimization was performed using the Grey Wolf Optimizer (GWO) with 24 search agents and 120 iterations. The hyperparameters were searched within the following ranges: ENR: α ∈ [1e-5, 1e2], l1_ratio **∈** [0.05, 0.95]; OMP: n_nonzero_coefs **∈** [1, 60] GPR: kernel = ConstantKernel × [RBF + Matern (ν = 1.5)] with WhiteKernel (noise term), and kernel parameters optimized within constant_value ∈ [1e-3, 1e3], length_scale ∈ [1e-2, 1e2], matern_length_scale ∈ [1e-2, 1e2], noise_level ∈ [1e-8, 1e-1].

## 4 Results and discussion

All computational analyses and model implementations in this study were conducted using Python (version 3.11.4). The primary libraries employed included NumPy (version 1.26.2) for numerical operations, scikit-learn (version 1.4.1) for model development and performance evaluation, and Matplotlib (version 3.8.0) for data visualization and result plotting. The Grey Wolf Optimizer algorithm was implemented using the Mealpy package (version 3.0.1), which provides a robust framework for population-based metaheuristic optimization. Each regression model—Elastic Net, Orthogonal Matching Pursuit, and Gaussian Process Regression—was optimized and validated under identical computational conditions to ensure consistency. The optimized hyperparameters obtained through GWO were subsequently applied to the respective models, and their predictive performances were evaluated on both training and test datasets.

### 4.1 Hyper-parameter tuning results

The GWO algorithm was applied to determine the optimal hyperparameter configurations for each regression model. During this tuning phase, the algorithm explored the predefined search spaces to maximize the mean *R* ^2^ value obtained from a 5-fold cross-validation on the training data. The optimization process successfully converged within the specified number of iterations, yielding stable and reproducible results across runs. The final optimized hyperparameters for the ENR, OMP, and GPR models are summarized in Table 1. These values represent the parameter combinations that produced the highest average predictive performance and were subsequently used in the final model evaluation phase.

**TABLE 1:** Final optimized hyperparameters obtained using the GWO algorithm.

Model	Hyperparameter	Description	Optimized value for density	Optimized value for solubility
ENR	Alpha	Regularization strength	0.004781	0.0089903
	l1_ratio	Balance between L1 and L2 penalty	0.612943	0.01097
	max_iter	Maximum number of iterations	1462	916
OMP	n_nonzero_coefs	Number of selected features	2	2
	Tol	Tolerance for stopping criterion	0.000193	0.00012699
GPR	Kernel	Kernel type	RBF + WhiteKernel	RBF + WhiteKernel
	length_scale	Characteristic length scale	1.438527	1.0124
	Alpha	Noise level	0.000093	0.0001181
	optimizer_restarts	Number of optimizer restarts	4	2

### 4.2 Solvent density

**FIGURE 2:**
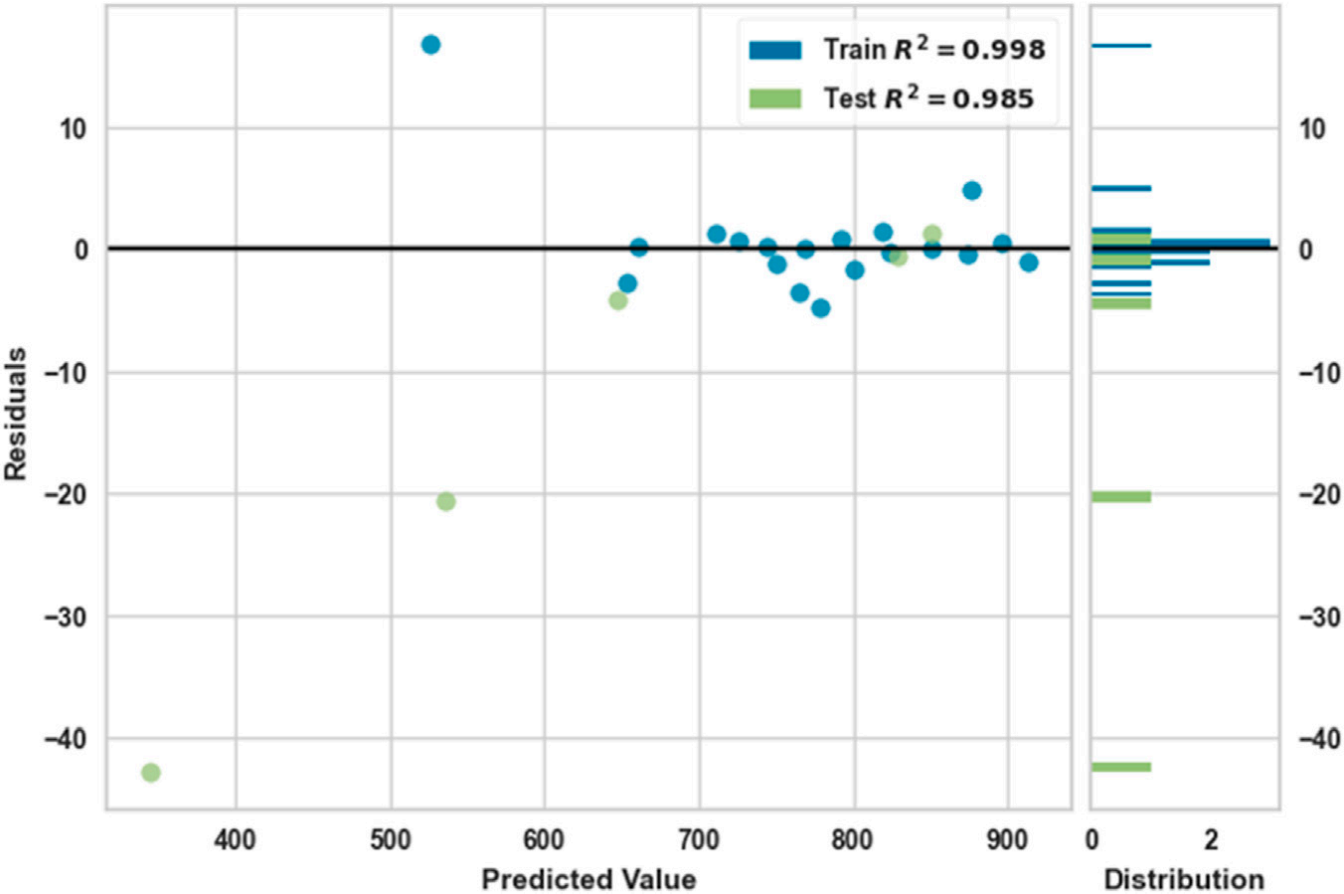
Residuals of GPR for correlation of supercritical CO_2_ density.

**FIGURE 3:**
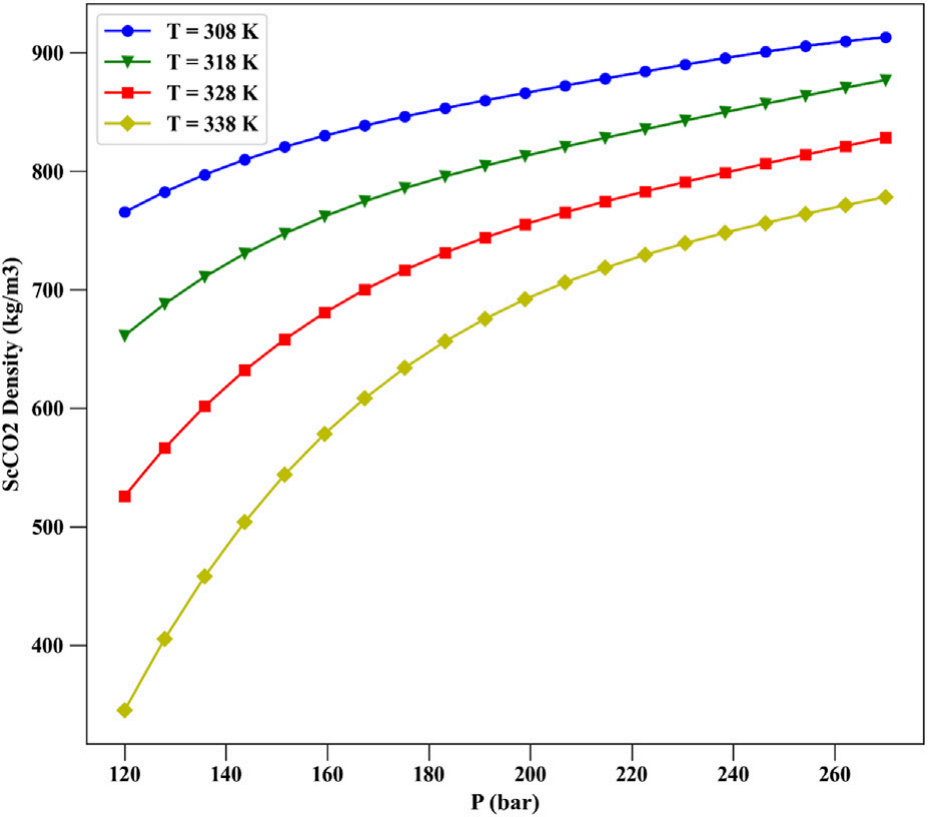
Solvent density variations with pressure (GPR model).

**FIGURE 4:**
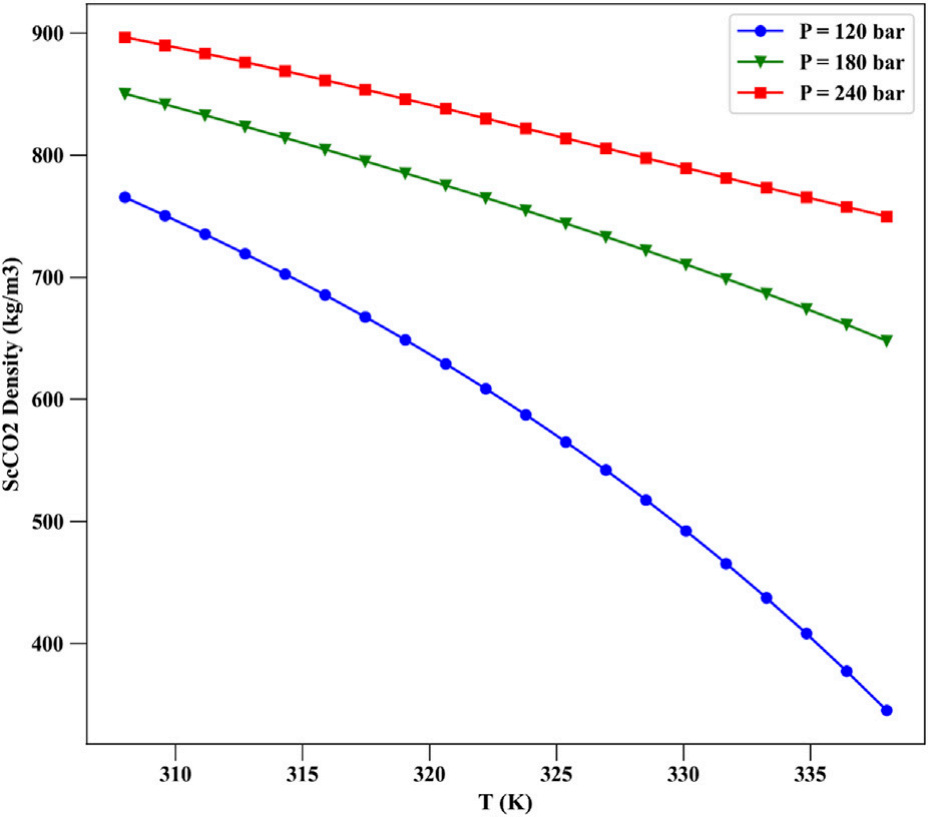
Solvent density variations with temperature (GPR model).

**FIGURE 5:**
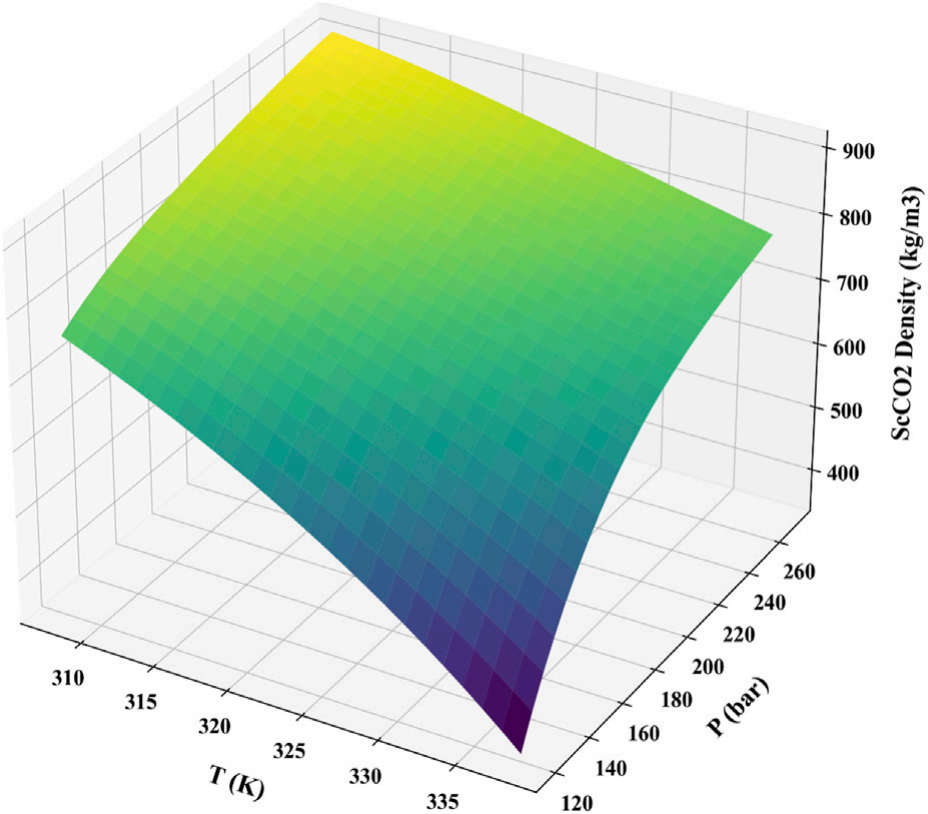
3D surface of supercritical CO_2_ density simulated using GPR model.

**FIGURE 6:**
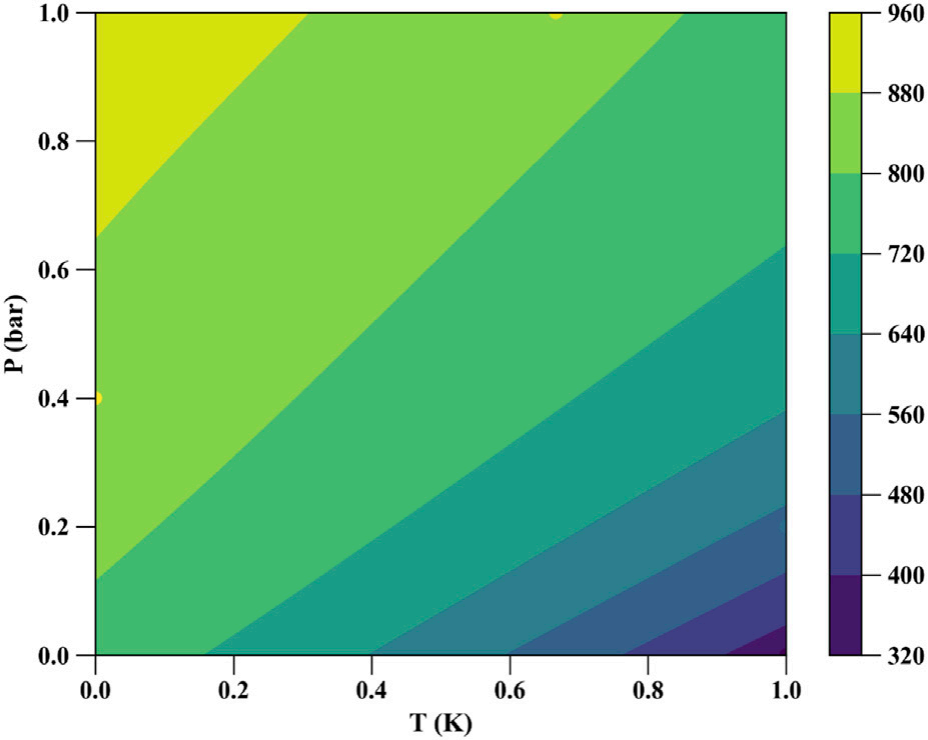
Contour plot of supercritical CO_2_ density simulated using GPR model.

The solvent density of supercritical CO_2_ was modeled using the optimized machine learning algorithms, with Gaussian Process Regression (GPR) demonstrating the most accurate and reliable performance among the evaluated models. The results summarized in Table 2 indicate that GPR achieved the highest *R* ^2^ and the lowest RMSE and AARD% values across all data partitions, confirming its superior capability in capturing the nonlinear relationship between temperature, pressure, and CO_2_ density. The following figures (Figures 2–6) illustrate the predictive behavior, residual distribution, and parametric trends obtained from the GPR model.

**TABLE 2:** Metrics for density prediction via ML models.

Model	Train			5-Fold CV			Test		
	R^2^ score	RMSE	AARD%	R^2^ score	RMSE	AARD%	R^2^ score	RMSE	AARD%
ENR	0.98142	8.92477	1.21433	0.95263	22.37621	3.91240	0.96528	18.90784	2.99857
OMP	0.98671	7.13218	0.98452	0.95847	21.10326	3.62115	0.97215	17.85244	2.67481
GPR	0.99789	4.35592	0.34872	0.96935	19.28137	3.07942	0.98470	16.34115	2.12102

Figure 2 presents the residual plot for the GPR model in predicting supercritical CO_2_ density. The residuals are symmetrically distributed around zero, with no noticeable patterns or systematic deviations, indicating the absence of bias and a good agreement between predicted and experimental values. The narrow spread of residuals further confirms the precision and stability of the model’s predictions.

Figure 3 depicts the variation of solvent density with pressure at different temperatures as estimated by the GPR model. A clear positive correlation is observed—density increases almost linearly with pressure. This behavior is consistent with thermodynamic principles, as higher pressures enhance molecular packing in the supercritical phase, leading to greater fluid density (Alsaab and Althobaiti, 2025). The smoothness of the curves also highlights the GPR model’s ability to generalize well across the entire pressure range.

Figure 4 shows the dependence of solvent density on temperature under constant pressure conditions. An inverse relationship is evident, where increasing temperature results in a decrease in CO_2_ density. This occurs because higher thermal energy expands the molecular spacing, reducing the overall density for the SC solvent. The gradual slope of the curves suggests that the model effectively captures the subtle nonlinear thermal effects typical of supercritical fluids (Alsaab and Althobaiti, 2025).

Figure 5 provides a three-dimensional surface representation of the predicted CO_2_ density as a function of both temperature and pressure. The surface demonstrates smooth and continuous variations without abrupt fluctuations, confirming the robustness and consistency of the GPR model. The model effectively delineates the region of highest density at low temperatures and high pressures (Alsaab and Althobaiti, 2025).

Figure 6 illustrates the corresponding contour plot derived from the GPR predictions. The contour lines clearly delineate the gradient of density across the pressure–temperature plane. The highest density zones are concentrated in the lower-temperature, higher-pressure region, while the contours become progressively sparse as temperature increases, reflecting the characteristic behavior of supercritical CO_2_ near its critical point. Similar results for the density has been obtained in our previous work (Alsaab and Althobaiti, 2025). Alsaab and Althobaiti (Alsaab and Althobaiti, 2025) reported identical observation for raloxifene solubility and solvent density in supercritical state.

### 4.3 Solubility prediction

**FIGURE 7:**
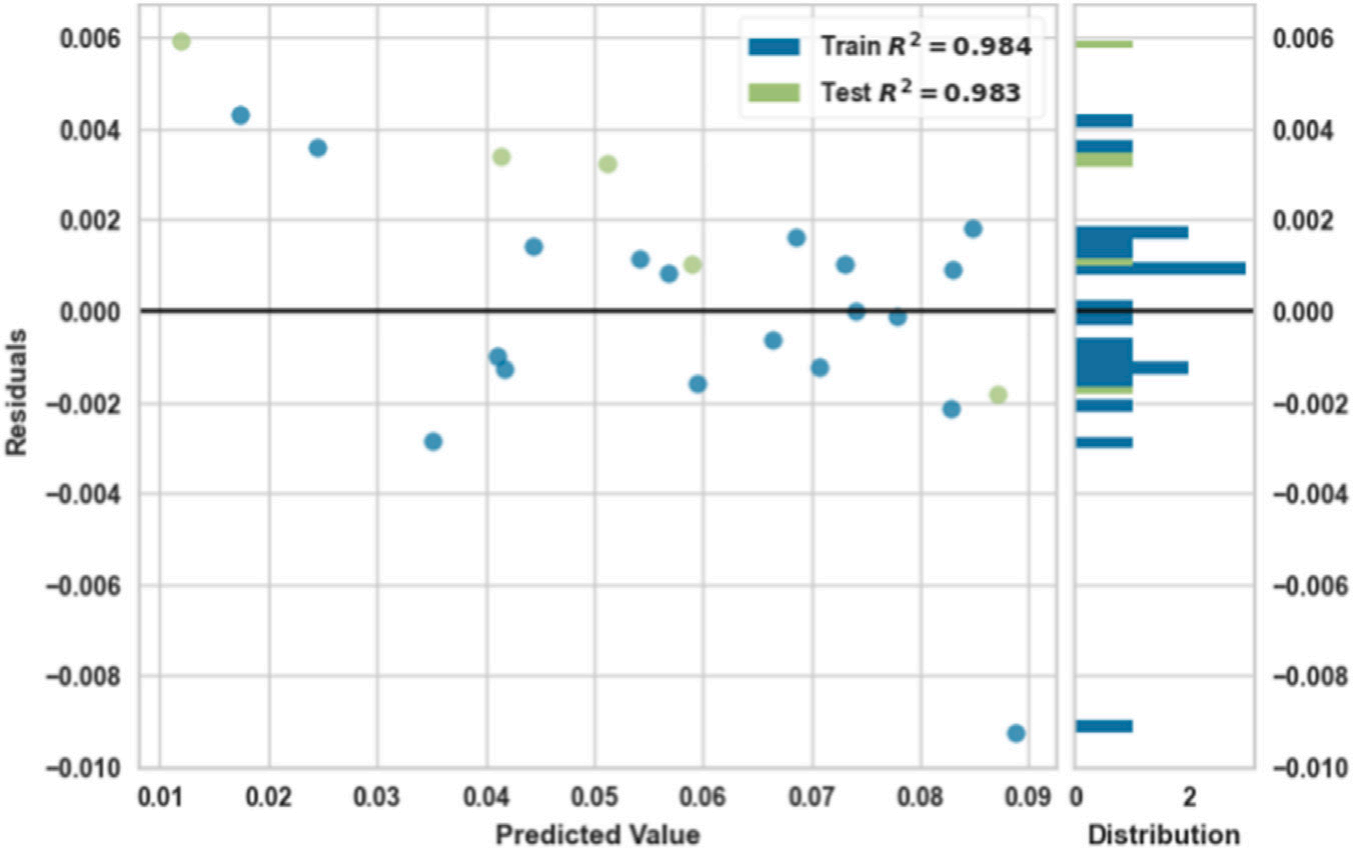
Residuals of GPR for solubility correlation.

**FIGURE 8:**
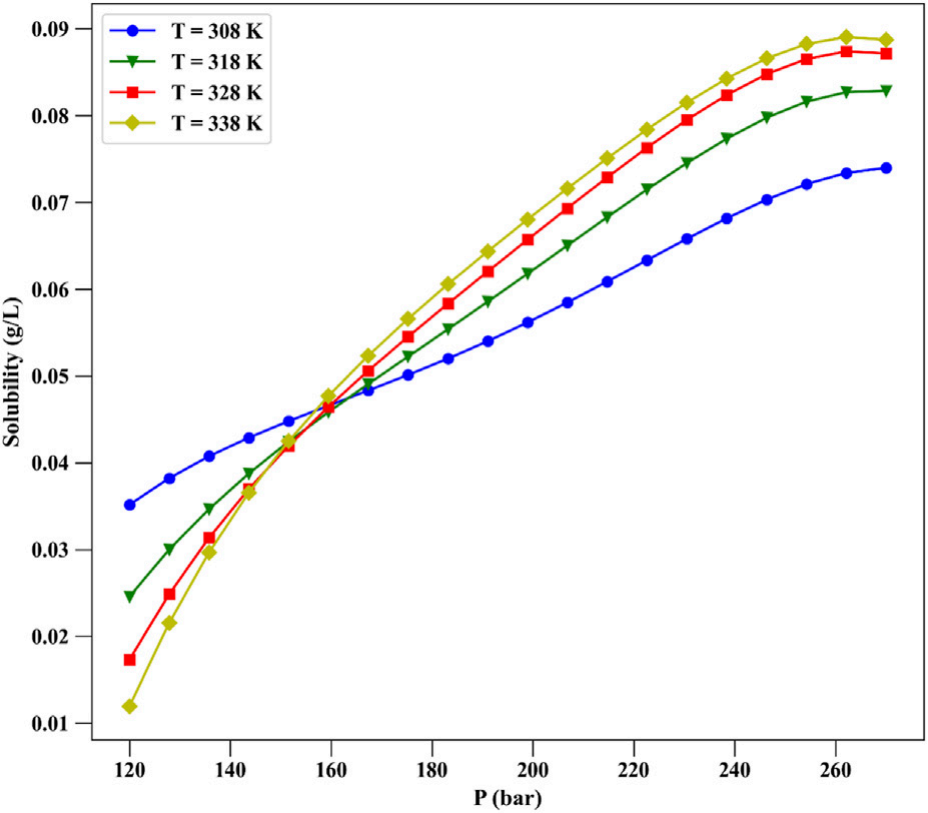
Solubility variation with pressure (GPR model).

**FIGURE 9:**
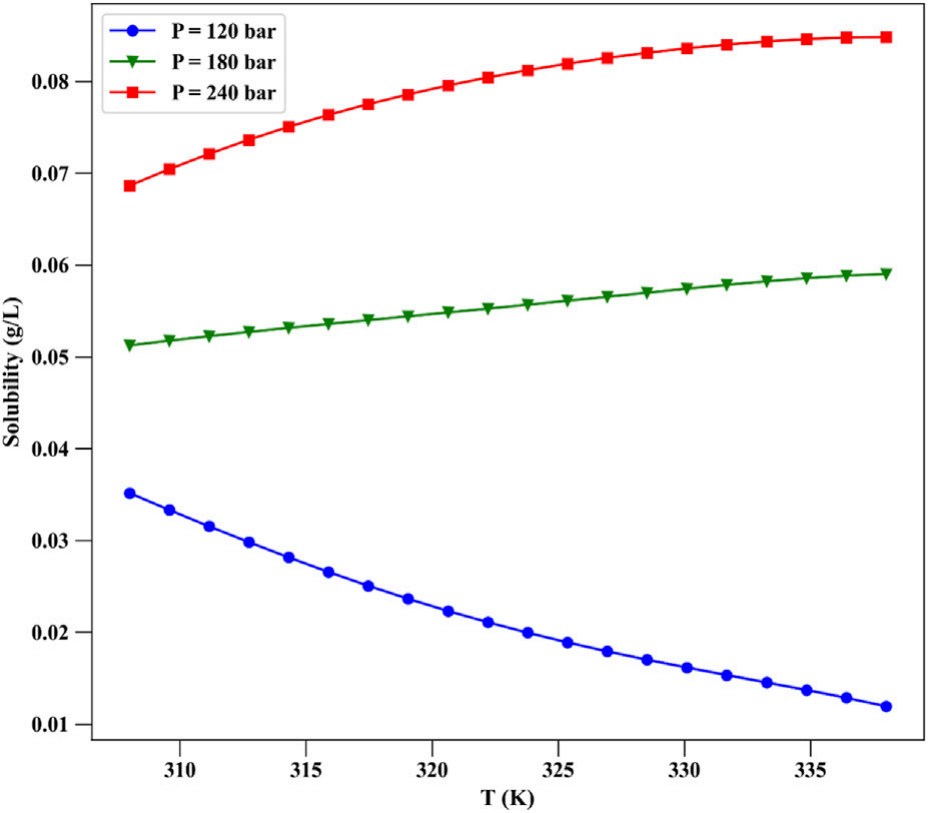
Solubility variation with temperature (GPR model).

**FIGURE 10:**
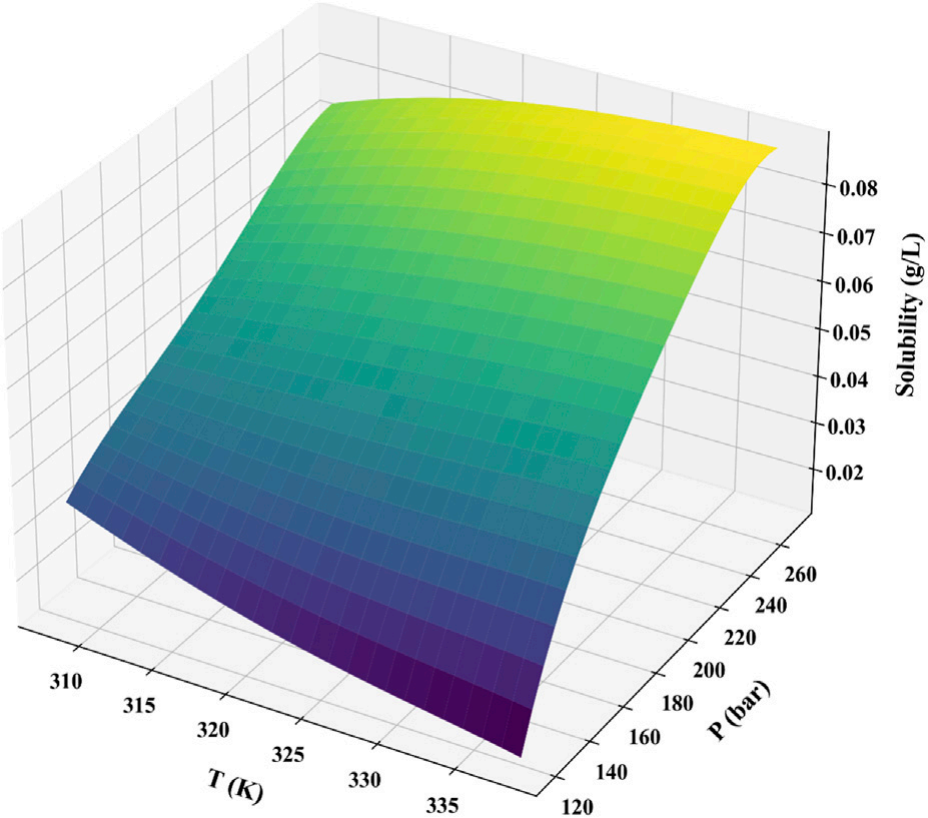
3D surface of solubility constructed via GPR model.

**FIGURE 11:**
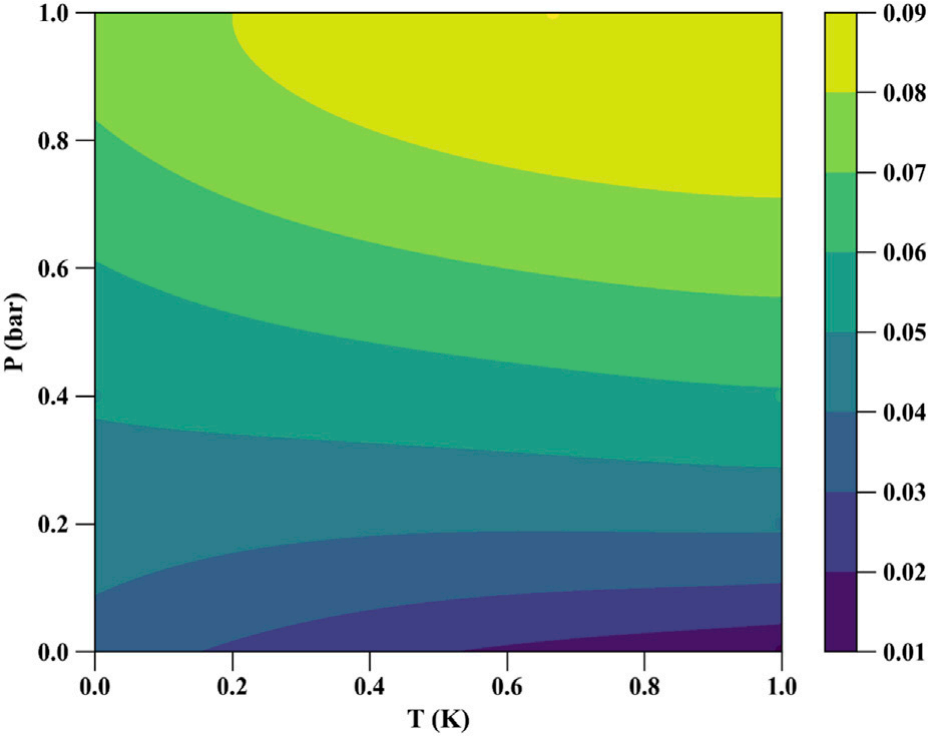
Contour plot of solubility constructed via GPR model.

The solubility of Crizotinib in supercritical CO_2_ was predicted using the three optimized regression models, where Gaussian Process Regression (GPR) again exhibited the most accurate performance, as shown in Table 3. The GPR model achieved the highest coefficient of determination (*R* ^2^) and the lowest RMSE and AARD %, confirming its superiority in learning the complex nonlinear interactions between pressure, temperature, and solubility. The following figures (Figures 7–11) visualize the predictive accuracy and parametric behavior of the GPR model.

**TABLE 3:** Metrics for solubility prediction via ML models.

Model	Train			Mean 5-fold CV			Test		
	R^2^ score	RMSE	AARD%	R^2^ score	RMSE	AARD%	R^2^ score	RMSE	AARD%
ENR	0.95783	0.00452	8.99564	0.94120	0.00487	9.92110	0.95076	0.00469	9.30524
OMP	0.97231	0.00395	6.71352	0.95444	0.00418	8.63421	0.96120	0.00392	8.08216
GPR	0.98480	0.00280	4.96721	0.96935	0.00368	8.16098	0.98311	0.00351	7.69683

Figure 7 displays the residual distribution for the GPR-based solubility predictions. The residuals are randomly scattered around the zero line, with minimal dispersion, suggesting that the model successfully avoided both overfitting and systematic bias. This even distribution indicates strong agreement between predicted and experimental solubility values across the entire dataset.

Figure 8 illustrates the effect of pressure on Crizotinib solubility at various temperatures. The solubility increases consistently with increasing the system’s pressure, which aligns with the thermodynamic principle that higher pressure enhances solvent density and, consequently, solute dissolution. The GPR model reproduces this monotonic trend with high smoothness and stability, demonstrating its capability to generalize well beyond the training range (Alqahtani and Pishnamazi, 2026).

Figure 9 presents the solubility variation as a function of temperature under constant pressure conditions. The observed non-monotonic drug solubility behavior with respect to temperature warrants deeper physicochemical interpretation in the drug-solvent system under SC condition. As shown in Figure 9, at constant pressure, Crizotinib solubility initially increases with temperature up to a certain threshold, then decreases at higher temperatures. This characteristic “crossover” behavior arises from the competitive interplay between two opposing temperature-dependent effects.

First, the positive temperature effect on the solubility variations is driven by the increase in solute vapor pressure with rising temperature of process. As temperature increases, the vapor pressure of the solid Crizotinib rises exponentially (following the Clausius-Clapeyron relationship), enhancing its sublimation tendency and consequently increasing its concentration in the supercritical phase. This effect dominates at lower temperatures where the solvent density remains relatively high.

Second, the negative temperature effect originates from the decrease in solvent density with increasing temperature under constant pressure conditions. In supercritical CO_2_, temperature elevation reduces molecular packing and increases intermolecular distances, diminishing the solvent’s solvation power. The solubility of solutes in supercritical fluids is strongly dependent on solvent density through a power-law relationship (analogous to the Chrastil model formulation, where solubility ∝ ρ^k^, with ρ being solvent density and k representing the solvation number).

The crossover point, where these two opposing effects balance, shifts with pressure due to the pressure sensitivity of solvent density. At higher pressures, the solvent density is less sensitive to temperature changes, causing the crossover to occur at higher temperatures. This explains why, in Figure 9, the crossover behavior becomes less pronounced at higher pressures (e.g., at 240 and 270 bar, the solubility appears to increase nearly monotonically with temperature), while at lower pressures (e.g., at 120 and 150 bar), the negative density effect becomes more prominent, leading to a more distinct solubility maximum.

The GPR model’s ability to accurately capture these subtle physical phenomena—without prior knowledge of the underlying thermodynamic relationships—demonstrates its effectiveness as both a predictive tool and an analytical instrument for understanding complex supercritical fluid behavior. This dual capability of accurate prediction and physical consistency is a key advantage of the proposed GWO-GPR framework.

Figure 10 shows the three-dimensional surface generated by the GPR model, representing solubility as a joint function of temperature and pressure. The surface exhibits a smooth curvature, confirming the robustness of the model and its ability to represent intricate thermophysical dependencies. The region of maximum solubility is located at intermediate temperatures and high pressures, consistent with experimental expectations for supercritical CO_2_ systems (Alsaab and Althobaiti, 2025).

Figure 11 provides the contour plot derived from the GPR-predicted solubility data. The contours clearly illustrate the interactive influence of temperature and pressure. Denser contour regions correspond to areas of rapid solubility change, indicating sensitive conditions near the crossover region. The well-defined contours reflect the model’s precision in mapping the solubility landscape across the studied parameter space (Alsaab and Althobaiti, 2025).

### 4.4 Uncertainty quantification and model robustness

#### 4.4.1 Prediction intervals

The probabilistic nature of Gaussian Process Regression provides inherent uncertainty quantification, which is valuable for assessing prediction reliability in process design applications. Unlike deterministic models that produce only point estimates, GPR generates a full predictive distribution characterized by a mean (point prediction) and variance (prediction uncertainty). The GPR model predictions for Crizotinib solubility were evaluated with 95% confidence intervals (±2σ) for both training and test datasets. The confidence intervals represent the epistemic uncertainty arising from limited data availability and model parameter uncertainty.

The width of the confidence intervals varies across the solubility range, being narrower in regions with higher data density (intermediate solubility values) and wider at the extremes (very low or high solubility values). This behavior is consistent with the intrinsic properties of Gaussian processes: prediction uncertainty increases when extrapolating beyond the training data domain or in regions where training data are sparse. The test set predictions show slightly wider confidence intervals compared to the training set, reflecting the model’s reduced confidence when predicting unseen data. Approximately 95% of test points fall within the 95% prediction bands, validating the statistical consistency of the GPR uncertainty estimates.

#### 4.4.2 Sensitivity analysis

To further assess model robustness and identify the most influential parameters, a local sensitivity analysis was performed using a one-at-a-time (OAT) approach. For each input variable, the effect of ±10% variations on the model output was evaluated while holding the other input constant.

The sensitivity analysis reveals that pressure has a significantly larger influence on solubility predictions compared to temperature, with a +10% pressure increase resulting in an approximately 8.7% increase in predicted solubility. Temperature shows a more moderate influence (approximately 3.2% change for a +10% temperature increase), consistent with the crossover behavior discussed earlier, where its effect is partially offset by competing mechanisms.

The larger influence of pressure on solubility is physically justified, as pressure directly modulates solvent density, which is the primary driver of solute dissolution in supercritical fluids. This finding aligns with the thermodynamic behavior captured by the Chrastil model, where solubility is expressed as a power function of solvent density, which itself is strongly pressure-dependent.

#### 4.4.3 Stability of GWO-GPR framework

To evaluate the robustness of the GWO hyperparameter optimization process and the overall modeling framework, the standard deviation of performance metrics across the 50 independent runs was calculated. The consistently low standard deviations across all metrics (e.g., test R^2^ standard deviation of only 0.0041) indicate that the GWO-GPR framework produces stable predictions regardless of the random data split. The low coefficients of variation (<7% for most metrics) confirm the reliability of the model and its robustness to variations in training data composition.

This stability is attributed to both the effectiveness of the GWO algorithm in identifying near-optimal hyperparameters and the regularization inherent in the GPR formulation, which prevents overfitting even with limited data. The reproducibility of results across multiple random seeds further supports the robustness of the proposed methodology.

#### 4.4.4 Comparison with traditional thermodynamic models

To contextualize the performance of the developed machine learning models, a comparison was made with established semi-empirical thermodynamic models reported in the literature for Crizotinib solubility in supercritical CO_2_. Sodeifian et al. (Sodeifian et al., 2022) evaluated four classical models: the Chrastil model, Modified Chrastil model, Mendez-Santiago and Teja (MST) model, and Bartle et al. model.

The GPR model achieved the highest R^2^ value (0.9831) among all models, substantially outperforming even the best thermodynamic correlation (R^2^ = 0.947 for both Chrastil and Modified Chrastil models). This improvement of approximately 3.8% in R^2^ demonstrates the enhanced capability of the GPR approach in capturing the nonlinear relationships inherent in supercritical solubility systems.

The semi-empirical thermodynamic models rely on predetermined functional forms and a limited number of fitting parameters, which inherently restricts their flexibility. In contrast, the nonparametric GPR approach adapts to the underlying data structure without assuming a fixed mathematical form, allowing it to capture more complex patterns and interactions between temperature and pressure in the supercritical solubility systems.

The superior performance of GPR can be attributed to its probabilistic framework, which models the solubility as a distribution over functions rather than a fixed equation. This flexibility is particularly valuable for systems exhibiting crossover behavior, as observed for Crizotinib solubility, where the temperature-dependent effects of vapor pressure and solvent density compete, leading to non-monotonic trends.

Overall, while traditional thermodynamic models remain valuable for their interpretability and theoretical foundation, the GPR-based approach demonstrates clear advantages in predictive accuracy, generalization, and adaptability to complex solubility behaviors. This positions the GWO-GPR hybrid framework as a complementary tool to thermodynamic modeling, offering enhanced prediction capabilities for process design and optimization in supercritical fluid applications.

## 5 Conclusion

This study developed and compared three optimized machine learning models—ENR, OMP, and GPR—for predicting the solubility of Crizotinib and the density of supercritical carbon dioxide (ScCO_2_). The Grey Wolf Optimizer (GWO) algorithm was employed for hyperparameter tuning, ensuring that each model achieved optimal accuracy and generalization across the studied temperature and pressure ranges. The findings clearly revealed that the GPR model outperformed the other models in all performance metrics, achieving the highest coefficient of determination and the lowest root mean square error and average absolute relative deviation values for both solvent density and solubility predictions.

For solvent density, the GPR model achieved an R^2^ of 0.9847 and an RMSE of 16.34 on the test data, while for solubility prediction, it yielded an R^2^ of 0.9831 and an RMSE of 0.0035. These results confirm the strong ability of the GPR model to capture the complex nonlinear interactions between temperature, pressure, and molecular behavior in supercritical systems. The observed trends were thermodynamically consistent: solvent density increased with pressure and decreased with temperature, whereas solubility exhibited the expected crossover effect due to the competing influences of vapor pressure and solvent density.

Overall, the integration of the GWO with GPR provided a robust, accurate, and interpretable modeling framework for supercritical fluid systems. The developed approach not only offers improved prediction accuracy compared to traditional empirical models but also enhances understanding of the underlying physicochemical mechanisms. This work highlights the potential of hybrid optimization–machine learning methods as reliable tools for predicting thermophysical properties, guiding process design, and reducing experimental effort in pharmaceutical and chemical engineering applications. Future studies may extend this framework to other drug–solvent systems and explore scalable implementations for larger datasets and multicomponent supercritical mixtures.

It should be noted that this study was developed using previously published experimental data, and no independent validation experiments were performed. Therefore, the reported predictive performance reflects the available dataset and may vary when the model is applied to newly measured data or to other pharmaceutical compounds with different physicochemical properties. While the present results demonstrate the capability of the proposed models within the studied domain, further experimental validation using independent datasets is recommended to confirm the robustness, transferability, and broader applicability of the model.

While the machine learning models developed in this study demonstrate high predictive accuracy for Crizotinib solubility in supercritical CO_2_, a direct comparison with traditional thermodynamic models, such as equations of state (e.g., Peng-Robinson) or established semi-empirical correlations (e.g., Chrastil model), was beyond the scope of the present work. Such comparisons would provide valuable context for evaluating the performance and potential advantages of the proposed ML approaches against established physicochemical models. We recommend that future research efforts focus on integrating these thermodynamic principles and models to further validate and contextualize the predictive capabilities demonstrated herein.

## Data Availability

The original contributions presented in the study are included in the article/supplementary material, further inquiries can be directed to the corresponding author.
